# Extracellular Vesicles in Epstein-Barr Virus’ Life Cycle and Pathogenesis

**DOI:** 10.3390/microorganisms7020048

**Published:** 2019-02-11

**Authors:** Mengmeng Zhao, Asuka Nanbo, Lichun Sun, Zhen Lin

**Affiliations:** 1 Tulane University Health Sciences Center and Tulane Cancer Center, New Orleans, LA 70112, USA; mzhao8@tulane.edu; 2 Graduate School of Medicine, Hokkaido University, Sapporo, Hokkaido 060-8638, Japan; nanboa@med.hokudai.ac.jp; 3 Department of Medicine, Peptide Research Laboratories, Tulane University Health Sciences Center, New Orleans, LA 70112, USA; lsun@tulane.edu

**Keywords:** Epstein-Barr virus, EBV, extracellular vesicle, EV, exosome

## Abstract

Extracellular vesicles (EVs), including exosomes and microvesicles, are evolutionarily conserved phospholidpid membrane-bound entities secreted from most eukaryotic cell types. They carry bioactive cargos such as protein and nucleic acids derived from their cells of origin. Over the past 10 years, they have been attracting increased attention in many fields of life science, representing a new route for intercellular communication. In this review article, we will discuss the current knowledge of both normal and virally modified EVs in the regulation of Epstein-Barr virus (EBV)’s life cycle and its associated pathogenesis.

## 1. Introduction

Extracellular vesicles (EVs) are a class of evolutionarily conserved membrane vesicles that are actively released by cells into the extracellular environment [1]. In normal physiological conditions, EVs can be secreted from most eukaryotic cell types including epithelial cells [2], endothelial cells [3], B cells [4], T cells [5], dendritic cells [6], smooth muscle cells [7], neurons [8], astrocytes [9], oligodendrocytes [10], and reticulocytes [11]. The actual list is still expanding at a fast pace. Under pathological conditions, abnormal cells such as cancer cells and virus-infected cells can also produce EVs for their benefit [12,13]. *In vivo*, EVs can be detected in various normal and pathological body fluids such as serum [14], plasma [15], semen [16], urine [17], saliva [15], amniotic fluid [18], cerebrospinal fluid [19], breast milk [15], bronchial alveolar lavage [20], and ascites [21], indicating that EVs can possibly reach any anatomic location/space inside the human body.

Based on their mode of production, EVs can be classified into three major groups: exosomes, microvesicles, and apoptotic bodies [13]. Exosomes (30–150 nm in diameter) originate from the inward budding of the endosomal membrane and are released by the fusion of multivesicular bodies (MVBs) and plasma membranes. Microvesicles (50–1000 nm in diameter) are generated by the outward budding of membrane vesicles directly from plasma membranes. Apoptotic bodies (800–5000 nm in diameter) are formed through the apoptotic pathway and released directly from the plasma membrane of apoptotic cells.

Currently, ultracentrifugation and density gradient centrifugation are the most common methods to purify the EVs. However, due to the inherent limitations of these methods, it is almost impossible to isolate pure EV subtypes. For instance, since exosomes and microvesicles share a partially overlapped size range, the isolated exosomes are inevitably mixed with microvesicles if using centrifugation-based purification techniques. Thus, for EV research, we need to keep in mind that the observed phenotypes are likely contributed to by more than one EV species.

Since almost all the EV research conducted in the EBV field focuses on small EVs such as exosomes, in this review we will mainly discuss what we know about exosomes in EBV’s life cycle and associated diseases. When exosomes were first discovered ~40 years ago, they were treated as an artifact and ignored by the research field [22]. Nowadays, we understand that exosomes are important messengers carrying various bioactive components including proteins, lipids, carbohydrates, and nucleic acids from their cells of origin. These biological cargos not only influence the local microenvironment, but also take effects far away from the source cells through circulation. Interestingly, evidence shows that exosomes can even cross the blood-brain barrier, and deliver their cargos to brain cells within highly secured anatomical regions [23]. Exosomes are a type of multi-functional entities. They have been shown to modulate many biological and pathological processes, such as carcinogenesis, cell proliferation, angiogenesis, immunoevasion, and metastasis [24]. Recently, the roles of exosomes in virus infection and virus-associated cancers have attracted lots of attention. Significant advances have been made in the field of EBV and exosome research.

## 2. Role of EVs/Exosomes Secreted from Healthy Cells in EBV’s Life Cycle

Epstein-Barr virus (EBV) is arguably one of the most successful human viruses. It elaborately evades the host immune surveillance and causes a life-long persistent infection of more than 90% of the adult population worldwide [25]. EBV mainly utilizes epithelial cells and B cells to fulfill its life cycle. Clinically, EBV is the first identified human tumor virus and is responsible for ~1.8% of all human cancers [25]. EBV plays important etiological roles in a number of human malignancies, including non-Hodgkin’s and Hodgkin’s lymphoma, nasopharyngeal carcinoma, and gastric carcinoma, as well as non-malignant diseases such as infectious mononucleosis [25].

Just like other members in the herpesvirus family, EBV demonstrates a biphasic life cycle that includes a latency phase and a lytic phase (replicative phase) [26]. After initial infection, EBV preferentially remains latent in host B cells without viral production. However, under certain circumstances, latency in B cells can be disrupted and the virus can shift into a productive viral replication phase [26]. The switch from latency to the lytic productive phase in B cells is a critical step of the virus life cycle that is important for virus persistence and pathogenesis. In the past, researchers have been looking for the endogenous signals that trigger the lytic cycle. Probably the most physiologically relevant signals are transforming growth factor β (TGF-β) and B-cell receptor (BCR) engagement [27,28,29]. However, although these signals show good *in vitro* data, it is still debatable if they are the *bona fide* signals to trigger virus production *in vivo* [26].

In the human body, the oral cavity functions as the factory for virus production and salivary virions are generated and spread among hosts through salivary exchange [30]. It is well known that latently infected B cells function as virus reservoirs to maintain a persistent infection. Unlike in B cells, EBV preferentially undergoes lytic replication in the epithelial tissues instead of latency [31,32]. The pro-lytic propensity of epithelial cells is likely to facilitate the production of high viral titers in the oral epithelium that can be released into the saliva. The B cell-to-epithelium transfer of EBV is mediated through the reactivating B cells that infiltrate the oral epithelium. We reasoned that the spontaneous viral reactivation in B cells is unlikely to be an efficient way to facilitate the B cell-to-epithelial-cell exchange process and epithelium-derived transmissible agents capable of inducing reactivation in B cells might actually help this process. The agent should act to trigger viral reactivation in B cells when the optimal environment (epithelium) is detected.

We and others previously reported that miR-200 family microRNAs (miRNAs) can act as a cellular switch that regulates the transition from latency to the lytic cascade [33,34]. Specifically, the miR-200 family miRNAs form a double negative-feedback loop with the ZEB1/ZEB2 (zinc finger E-box binding homeobox 1 and 2) lytic cycle repressors, to facilitate the committed transition of cells between the latent and lytic cycles [33,34,35,36]. Increasing data have shown that miRNAs are important cargos in exosomes and they can alter the signaling pathway in recipient cells [37]. In agreement with it, we showed that the oral epithelium-associated EV pathway, especially the exosome plays a key role in orchestrating EBV’s lytic replication in B cells [2]. In circulation or other non-epithelial environments, the low levels of endogenous miR-200 family miRNAs in EBV-infected B cells allow the expression of lytic suppressors ZEB1/ZEB2 and inhibit the viral lytic activator Zta (Z transactivator) gene, thus promoting viral latency. In the epithelial environment, the epithelial markers, miRNA-200 family miRNAs, are actively loaded into small EVs including exosomes. Once released, these exosomes create an epithelial microenvironment that favors EBV lytic replication. The internalization of exosomal miR-200s in recipient B cells will subsequently activate lytic replication by relieving ZEB1/ZEB2 mediated suppression of the viral Zta gene. We concluded that miR-200s are ideal exosomal signaling molecules for facilitating sustained pathway alterations. This self-reinforcing feedback mechanism allows a single dose of exosomal miR-200s to not only inhibit ZEB1/ZEB2 expression, but also increase cellular miR-200 expression and help break viral latency in recipient cells [2]. With the assistance of exosomes, EBV stored in the peripheral B cells can be amplified in the oral epithelium and subsequently shed into the saliva. Thus, this microenvironment sensing mechanism can help facilitate the virus exchange between B cells and epithelial cells. Further, it also enables such a limited number of virally infected B cells (~ one in one million circulating B cells) to maintain constant viral replication foci at oral epithelial sites.

Notably, we have focused on one-way communication from oral epithelial cells to virally infected B cells [2]. It is also interesting to consider whether in the microenvironment, the virally infected B cells can simultaneously “talk to” oral epithelial cells through an exosome-mediated paracrine-like manner and modulate signal pathways that may facilitate the needs of the virus. For instance, uptake of these exosomes may induce the permissiveness of recipient epithelial cells to EBV infection. In agreement with this hypothesis, Nanbo et al. showed that uptake of exosomes released from EBV(+) B cells enhances the expression of intercellular adhesion molecule-1 (ICAM-1) in epithelial cells, which subsequently facilitates cell-to-cell contact-mediated EBV transmission [38,39]. Further, a recent study from Nanbo’s group has shown that suppression of exosomes biogenesis in either EBV(+) B cells or uninfected epithelial cells significantly impairs the EBV transmission between B cells and epithelial cells, indicating an important role of exosomes in the regulation of EBV’s infection cycle [40].

## 3. EBV-Modified Exosomes and Their Roles in EBV’s Life Cycle and Pathogenesis

To date, various EBV gene products have been detected in the exosomes released from virally infected cells. However, due to the technical limitations, it is unclear if these viral contents co-exist in a single exosome or each viral factor is associated with an individual subpopulation of exosomes.

### 3.1. EBV miRNA(+) Exosomes

MiRNAs are single-stranded non-protein coding regulatory RNAs with a length of ~22 nucleotides. EBV is the first virus known to carry viral miRNA genes. It encodes at least 44 mature miRNAs which are processed from 25 miRNA precursors [41]. These miRNAs are derived from two distinct clusters within the viral genome. Three EBV miRNAs are encoded within the BamHI H rightward open reading frame (BHRF) region and the remaining miRNAs are located within the BamHI A rightward transcript (BART) region. There is accumulating evidence that these highly expressed viral miRNAs play critical roles in EBV’s life cycle and pathogenesis by promoting cell transformation, inducing cell proliferation, blocking apoptosis, and escaping from host immune surveillance [41,42,43,44].

Like cellular miR-200 family miRNAs, EBV-encoded miRNAs are loaded into exosomes. Pegtel et al. have shown that the mature forms of EBV BART and BHRF1 miRNAs are selectively enriched in exosomes secreted from EBV-infected lymphoblastoid cells (LCL) [45]. These exosomal viral miRNAs, including miR-BHRF1-3, miR-BART1-5p, miR-BART2-5p, and miR-BART3, can be internalized by co-cultured dendritic cells. Interestingly, in the recipient cells, the exosomal miR-BHRF1-3 silences one of its known cellular targets CXCL11 (C-X-C motif chemokine ligand 11), an immunostimulatory gene. Similarly, uptake of the exosomal miR-BARTs also represses LMP1 (latent membrane protein 1), one of their known targets, indicating a functional delivery of these viral miRNAs. Later on, having profiled the exosomal miRNA secreted from EBV(+) LCL cells, Gallo et al. found that all the expressed BART and BHRF1 miRNAs are sorted to the exosomes more or less [46]. A selective enrichment of miR-BART3 and miR-BHRF1-1 in the exosomes are also observed. Further, Haneklaus et al. has shown that the exosomal EBV miR-BART15 released from EBV-infected B cells can inhibit the host anti-viral activity by suppressing NLRP3 (NLR family pyrin domain containing 3) inflammasome-mediated IL-1β (interleukin-1β) production [47]. Taken together, these data suggest that exosomal EBV miRNA may function as a new messenger to facilitate intercellular communication and create an immune microenvironment tolerating EBV infection.

Generally, based on the EBV gene expression pattern, latent infection can be classified into 4 different types: Type 0, Type I, Type II, and Type III latencies. Type 0 latency can be found in quiescent memory B cells and is characterized by expression of EBV-encoded small RNAs (EBERs) and Bam HI A rightward transcripts (BARTs). Type I latency is associated with Burkitt’s lymphoma and expresses EBNA1 (EBV nuclear antigen 1), EBERs, and BARTs. Type II latency can be seen in the nasopharyngeal carcinoma with the expression of EBNA1, LMP1, LMP2s (latent membrane protein 2s), EBERs, and BARTs. Type III latency is associated with lymphoproliferative disease, in which the full repertoire of viral genes is expressed, including EBV nuclear antigens (EBNA1, 2, 3s, LP), LMP1, LMP2s, EBERs, and BARTs.

In a recent study, Nanbo and colleagues took advantage of next-generation sequencing technology to globally analyze the miRNA transcriptome in exosomes released from Burkitt’s lymphoma cells carrying different EBV latent infection types [48]. They found that both EBV BART and BHRF1 miRNAs were actively packed to the exosomes. Exosomes released from Type I latency infection and Type III latency infection cells show distinct profiles of viral exosomal miRNAs. Further, the Type III latency infection shows higher level of exosome production and greater concentration of exosomal viral miRNAs. The specific enrichment of viral miRNA in exosomes cannot be simply explained by the number of exosome loading motifs (EXOmotifs) carried by each viral miRNA and the Type III latency gene expression might also contribute to the specific loading of the viral miRNAs [48].

In accordance with it, Higuchi et al. have recently shown that EBV BART miRNAs are loaded to the exosomes released from EBV(+) Burkitt lymphoma cells [49]. Uptake of exosomal miR-BARTs can induce the immune regulatory phenotype in macrophages by upregulating the expression of tumor necrosis factor-α (TNF-α) and interleukin-10, as well as enhancing the cell viability. Moreover, both their *in vitro* and *in vivo* data support the notion that the miR-BART(+) exosomes can facilitate lymphoma development by establishing a macrophage-related pro-tumor inflammatory niche.

In another report, Meckes and colleagues have shown that EBV BART miRNAs (including miR-BART-1, 4, 5, 7, 9, 11, 12, 13, and 16) are also selectively enriched in exosomes secreted from the EBV positive nasopharyngeal carcinoma (NPC) cell cultures. These exosomal EBV miRNAs can be delivered into the uninfected cells [50]. Furthermore, in the *in vivo* settings, EBV miRNAs are also detected in the exosomes purified from NPC patient sera [51]. Among these exosome-associated BART miRNAs, miR-BART13-3p shows the highest enrichment [51]. Compared to the existing methods for NPC diagnosis including viral DNA load and IgA (immunoglobulin A) EBV serology, detection of the exosomal miR-BART13-3p shows a better diagnostic potential with a higher specificity and sensitivity. Thus, circulating exosome-bound BART-13-3p is likely a promising biomarker for non-invasive early diagnosis of NPC.

### 3.2. LMP1(+) Exosomes

Latent membrane protein 1 (LMP1) is expressed in the EBV Type II and Type III latency infection and it is a key viral oncogene involved in EBV-associated carcinogenesis by promoting transformation, invasion, angiogenesis, immunoevasion, and metastasis, as well as the Warburg effect [52,53,54]. The exosome-bound LMP1 was first reported by Dukers et al. in 2000 [55]. Using the ultracentrifugation method, they isolated the exosomes from EBV(+) B cell cultures and then confirmed the presence of LMP1 by the Western blot assay. It is believed that these LMP1(+) exosomes can function as immune suppressors to inhibit T cell activation and NK (natural killer) cell cytotoxicity. Three years later, Vazirabadi et al. reported that LMP1 can be detected in the small EVs released from the EBV(+) lymphoblastoid cell line [56]. In 2007, Houali and colleagues showed that LMP1 was present in exosomes isolated from both NPC cell cultures and serum of NPC patients [57]. Meanwhile, these LMP1(+) exosomes can enhance cell cycle progression in an LMP1-dependent manner. In the same year, Ceccarelli et al. discovered the co-existence of LMP1 and its induced angiogenic factor FGF-2 (fibroblast growth factor 2) in exosomes [58]. Uptake of these exosomes promotes the proliferation of blood vessel endothelial cells, indicating a potential role of these exosomes in the induction of angiogenesis in tumor development [58]. Later, Meckes et al. investigated the effects of LMP1 on the content and properties of exosomes secreted from NPC cells [50]. They found that LMP1 increases the loading of EGFR (epidermal growth factor receptor) into the exosomes. These LMP1(+) exosomes can be taken by various types of cells and then activate ERK (extracellular signal-regulated kinase) and PI3K/Akt (phosphoinositide 3-kinase/protein kinase B) signaling pathways in the recipient cells. Later on, Aga et al. reported that LMP1 also up-regulates the level of HIF1α (hypoxia-inducible factor-1α) in exosomes [59]. These LMP1(+) exosomes increases the migration and invasiveness of the recipient nasopharyngeal cell lines [59]. Together, these data suggest an important role of LMP1(+) exosomes in EBV infection and its associated diseases. Indeed, since it is believed that only a portion of infected tumor cells express LMP1 *in vivo*, exosome-mediated delivery may allow a limited number of LMP1(+) cells to modulate the tumor microenvironment and facilitate viral infection. To elucidate the mechanism controlling the biogenesis of these LMP1(+) exosomes, Kobayashi et al. reported that directing LMP1 to exosomes is mediated by ubiquitin c-terminal hydrolase-L1 (UCH-L1) and the c-terminal farnesylation of UCH-L1 acts as a key step to sort LMP1 to exosomes [60]. In addition, Hurwitz et al. showed that CD63 (cluster of differentiation 63) is also required for LMP1 sorting to exosomes and LMP1-induced exosome secretion [61,62].

### 3.3. LMP2A(+) Exosomes

Like LMP1, Ikeda et al. have shown that EBV latent membrane protein 2A (LMP2A) can be secreted through exosomes [63]. These exosomal LMP2A are ubiquitinated but not phosphorylated, suggesting that ubiquitination may be required for LMP2A loading into the exosomes. They also found that the biogenesis of LMP2A(+) exosomes is controlled by the cholesterol level in the original cells, since depletion of cholesterol from plasma membrane can drastically increase the secretion of LMP2A(+) exosomes. The biological role of these LMP2A(+) exosomes is still unknown. It is postulated that the exosomal LMP2A may mimic the B cell receptor signal and help facilitate the EBV life cycle by providing development and survival signals in the recipient cells.

### 3.4. EBERs(+) Exosomes

EBV actively expresses two non-protein-coding RNAs (EBER1 and EBER2) in almost all its infected cells [64]. These highly expressed polymerase III transcripts can interact with cellular proteins such as the double-stranded RNA-dependent protein-kinase R (PKR), the lupus antigen La, the retinoic acid inducible gene 1 (RIG-I), and the ribosomal protein L22 (rpL22) [65,66,67,68,69]. EBERs contribute to viral carcinogenesis by blocking apoptosis, enhancing proliferation, and promoting tumor formation. Ahmed et al. have reported that both EBER1 and EBER2 were detected in the exosomes purified from the EBV-infected cell lines [70]. The co-existence of EBERs and the EBER-binding protein La indicates that EBERs might be loaded into exosomes in the form of RNA-protein complex. In accord, both EBER1 and EBER2 were also detected in the exosomes released by EBV(+) LCL cells [71]. Nevertheless, although both EBERs were secreted out, only EBER1 can be detected in the recipient cells, and the fate of the exosomal EBER2 is still unknown. Functionally, the exosomal EBER1 can induce Interferon (IFN)-related genes and interleukin-6 in a cell-type dependent manner. To date, the biological properties of these EBER(+) exosomes and their roles in viral life cycle remain largely unclear.

### 3.5. Gp350(+) Exosomes

Vallhov et al. have shown that EBV late gene product glycoprotein 350 (gp350) can be actively loaded on the exosomes produced by the EBV(+) LCL cells [72]. Interestingly, these gp350(+) exosomes can selectively bind to B cells. The interaction is mediated by the gp350 on the exosome surface and CD21 (cluster of differentiation 21) receptor on the B cells. It is shown that these exosomes can inhibit EBV infection in the *in vitro* cell culture system. It is been postulated that the host cells may utilize these gp350(+) exosomes to control the spread of EBV infection by preventing subsequent infection of bystander B cells.

### 3.6. EBV mRNA(+) Exosomes

Besides viral small RNA and miRNAs, a study has recently shown that EBV protein-coding RNAs also exist in the virally modified exosomes [73]. Four key latent gene transcripts were detected, including LMP1, LMP2, EBNA1, and EBNA2. However, it is still unclear if these viral mRNA can be delivered into the recipient cells and if these transcripts are biologically active in the uninfected recipient cells.

### 3.7. EBV-Modified Exosomes Carrying Cellular Gene Products

It will be interesting to know whether unique cellular products can be selectively loaded into these virally modified exosomes. To elucidate the global effects of EBV on the exosome content, Meckes and colleagues carried out a high-throughput proteomics analysis of the exosomes released from EBV infected cells [74]. The results demonstrate that a large number of cellular proteins are uniquely loaded into the EBV-modified exosomes, indicating that EBV can efficiently hijack the host exosome pathway. Further, having done an Ingenuity pathway analysis, they found that key exosomal cargos such as integrins, actin, interferon, and nuclear factor (NF)-κB may regulate various biological processes including cell viability, protein synthesis, and ribosome function in recipient cells, which may contribute to EBV infection and pathogenesis.

In addition, Gallo et al. have examined the cellular long non-coding RNAs (lncRNA) packed in the exosomes released from EBV(+) LCL cells [46]. They found that majority of examined lncRNAs show lower levels in exosomes compared to the cellular fraction. Nevertheless, two cellular lncRNAs, H19 and H19 antisense are actively packed into exosomes, indicating a potential role of these two lncRNAs in the regulation of the EBV life cycle.

To date, the majority of studies utilize traditional methods such as microarray or PCR (polymerase chain reaction) to characterize the RNA components in the exosomes. The inherent limitation of these techniques will inevitably impair the accuracy (both specificity and sensitivity) of the results. Recently, Nanbo and colleagues have utilized a next generation sequencing (NGS)-based informatics approach to globally characterize the miRNA species within exosomes isolated from EBV(+) Burkitt’s lymphoma cells [48]. They found that a large number of cellular miRNAs exist in the EBV-modified exosomes. Further, the type of EBV latency infection functions as a major factor determining the abundance and types of secreted exosomal miRNAs.

## 4. Challenges and Future Directions

To date, emerging evidence supports the notion that both normal and virally modified EV/exosomes play critical roles in EBV’s life cycle and pathogenesis (Table 1). Nevertheless, the majority of the conclusions are drawn based on the results obtained in the *in vitro* cell systems. Thus, due to the limitation of the current model systems, it is still questionable if the observed phenotypes and predicted mechanisms truly occur *in vivo*. So far, there are only very limited studies showing the activities of EV/exosomes *in vivo*. To the best of our knowledge, there is no direct evidence showing that EVs/exosomes can regulate EBV’s life cycle and pathogenesis in the *in vivo* context. Lots of important questions remain unanswered. Recently, some groups develop promising tools to track EV/exosomes with traceable markers in their *in vivo* animal model [75,76,77]. These tools might eventually help us find answers to long-standing questions in the field: How abundant are these EVs/exosomes in the *in vivo* microenvironment? How do these EVs/exosomes recognize their target cells and deliver the cargos?

Meanwhile, due to the technical limitations, it is currently an impossible mission to purify/isolate one type of EV without contaminating other types of vesicles [78]. Thus, the observed phenotype is due to the overall effects of EV mixtures. In addition to vesicle contamination, the isolated EVs are also contaminated with large protein/RNA/DNA complexes [78,79,80]. For instance, lipoprotein-associated RNAs can be co-purified with EVs and these protein-bound RNAs are resistant to RNase treatment. Importantly, like EVs, such complexes can also be delivered to the recipient cells [80,81]. Thus, to minimize the contaminations, it has been recommended by research societies to use multiple isolation methods. For instance, to optimize the exosomes purification, one option is to use ultracentrifugation method in conjunction with density gradient method or antibody-based immune-isolation [82]. Meanwhile, to better elucidate the mechanism, it has been suggested to comprehensively analyze all the subtypes of EVs in the model systems and subsequently pinpoint which subtype plays a major role in determining the phenotypes.

At this moment, there is no good standard (size, structure, density, markers) to distinguish these subtypes. A better understanding of EVs will certainly help us develop more efficient methods to isolate each subtype. With the efforts of the whole community, new EV markers are continuously discovered, which may help us solve the isolation issues in the future [83].

Currently, EV-oriented EBV research is still in its infancy and a lot of questions remain unanswered. *In vitro*, it is still unclear how EBV regulates EV biogenesis and the content and function of EBV-modified EVs needs to be further investigated. There is much more we do not know about the interaction between EV and EBV in the context of the *in vivo* setting. However, with the advance of new techniques such as better EV tracking systems and a better understanding of EVs in general, we will certainly get better ideas of the exact role of EVs in EBV’s infection and pathogenesis, which is important for better control and management of EBV-associated diseases in the future. Indeed, the possibility of usage of the EV contents as potential biomarkers is currently being evaluated in the clinical setting. The unique viral contents carried by these EBV-modified EVs and the easy accessibility through body fluids make them a promising non-invasive tool for the early diagnosis and prognosis of various EBV-associated diseases such as cancers.

## Figures and Tables

**Table 1:** Extracellular vesicle (EV) cargos and their roles in the regulation of Epstein-Barr virus (EBV) infection and pathogenesis.

EV Cargos	Cargo Types	Biological Effects	References
miR-BARTs and miR-BHRF1s	EBV miRNAs	Regulate immune phenotype; promote carcinogenesis	[45,46,47,48,49,50,51]
EBERs	EBV small ncRNA	Induce IFN-related genes and IL-6; immune regulation	[70,71]
LMP1, LMP2, EBNA1, EBNA2	EBV mRNAs	Facilitate EBV infection and carcinogenesis?	[73]
LMP1	EBV Protein	Immune regulation; promote carcinogenesis	[55,56,57]
LMP2A	EBV Protein	Enhance cell viability?	[63]
Gp350	EBV protein	Recognize recipient cells; regulate EBV infection	[72]
miR-200 family miRNAs	Cellular miRNAs	Regulate EBV infection cycle	[2]
EGFR	Cellular Protein	Activate kinase signaling pathway; promote tumor development	[50]
PI3K	Cellular Protein	Activate kinase signaling pathway; promote tumor development	[50]
FGF-2	Cellular Protein	Promote angiogenesis; promote tumor development	[58]
HIF1a	Cellular Protein	Promote cell migration and invasiveness	[59]
UCH-L1	Cellular Protein	Sort LMP1 to Exosome	[60]
CD63	Cellular Protein	Sort LMP1 to Exosome	[61,62]
La	Cellular Protein	Sort EBERs to exosomes?	[71]
HLA class I and II	Cellular Protein	Facilitate viral entry and immune regulation?	[74]
